# Exploring the influence of leadership styles on psychological well-being and satisfaction of Pilates classes clients

**DOI:** 10.1186/s13102-024-00949-8

**Published:** 2024-07-23

**Authors:** Youngmi Kim, Soowon Chae, Insuk Sim, Buom Kim

**Affiliations:** 1 Department of Sports Science, University of Kyonggi, Suwon, 16227 Gyeonggi-do Republic of Korea; 2 Department of Social and Physical Education, University of Hansin, Osan, 18101 Gyeonggi- do Republic of Korea; 3 Department of Clinical Laboratory Science, Dongnam Health University, Suwon, 16328 Gyeonggi-do Republic of Korea; 4 Department of Physical Education, University of Kyungdong, Yangju-si, 11458 Gyeonggi-do Republic of Korea

**Keywords:** Pilates, Leadership styles, Class satisfaction, Psychological well-being, Fitness

## Abstract

**Background:**

The recent trend of increased indoor activities has significantly influenced daily life, enhancing the popularity of exercises like Pilates. This study explores how Pilates instructors’ leadership styles affect class satisfaction and psychological well-being, addressing the research gap concerning the specific impact of leadership within the context of Pilates classes.

**Methods:**

The study surveyed 388 participants from 39 Pilates studios across South Korea, utilizing a questionnaire to assess leadership styles (transformational, transactional, and servant leadership), class satisfaction, and psychological well-being. Using statistical analyses, leadership styles were assessed for their correlation with class satisfaction and psychological well-being.

**Results:**

Findings revealed that transformational and transactional leadership styles were positively correlated with class satisfaction and psychological well-being. Specifically, transformational leadership significantly enhanced educational satisfaction and personal growth, while transactional leadership most strongly influenced social satisfaction and the sense of purpose in life. Servant leadership was notably effective in improving physical class satisfaction. The study also highlighted the critical role of class satisfaction in promoting psychological well-being among participants.

**Conclusion:**

This research underscores the importance of leadership styles in enhancing the Pilates class experience, suggesting that instructors’ leadership approaches can significantly impact participants’ satisfaction and psychological well-being. The study advocates developing training programs that equip Pilates instructors with the skills to adopt effective leadership styles, fostering a more positive and fulfilling class environment.

**Supplementary Information:**

The online version contains supplementary material available at 10.1186/s13102-024-00949-8.

## Introduction

In recent years, there has been a notable shift towards more home-based physical activities, reflecting broader lifestyle changes [1, 2]. This trend includes a variety of exercises such as skipping rope, badminton, Pilates, and yoga, which have become increasingly popular due to their convenience and accessibility [3]. These activities cater to a wide demographic, particularly appealing to women who find these exercises adaptable to indoor environments [4]. The rising popularity of such exercises underscores a growing recognition of their benefits, which extend beyond physical health to include mental and emotional well-being [5, 6]. This shift towards home-based fitness routines, including the easy access to Pilates exercises from the comfort of one’s home, aligns with broader wellness trends and underscores a significant transformation in how people prioritize and engage in physical activity. This accessibility not only promotes the growth of indoor activities but also facilitates the expansion into various forms of exercises like Pilates [7, 8].

In this context, the satisfaction of participants in Pilates classes is significantly influenced by the attributes of instructors and staff. Research by Ghandali, Iravani, Habibi, and Cheraghian [9] suggests that the quality of Pilates instruction and participant satisfaction play pivotal roles in enhancing continued participation and fostering a fondness for exercise, thereby positively influencing the intention to re-engage.

Additionally, the formation of relationships between participants and the physical environment, coupled with the human services provided by instructors, has been identified as crucial, as evidenced by the studies of Liu, Zhu, and Jiang [10] and Rodrigues et al. [11].

The leadership of Pilates instructors is instrumental in controlling participant behavior, enhancing exercise functionality, and fostering psychological stability. Studies by Pappas, Panou, and Souglis [12] and F. Mavrovouniotis et al. [13] demonstrate that effective leadership in Pilates can significantly impact participant satisfaction and the intention to continue the exercise. Furthermore, research by Rowe and Slater [14] and Kayaoğlu and Özsu [15] indicates that the leadership style of Pilates instructors positively influences participants’ perceptions, values, and needs, thereby strengthening psychological elements and yielding favorable outcomes in exercise performance.

Factors influencing class satisfaction include participant motivation, instructor communication skills, willingness to participate, immersion, and physical transformation. Studies by Hosseini, Ghorbani, and Rezaeeshirazi [16] and Gholidahaneh, Ghorbani, and Esfahaninia [17] highlight the significance of instructor expertise, communicative ability, and environmental factors such as cleanliness and safety in elevating participants’ satisfaction with the class. Additionally, research by Santos et al. [18] has explored the impact of clinical Pilates on women experiencing urinary incontinence, suggesting that regular Pilates exercises can enhance women’s psychological well-being and life satisfaction.

Leadership in a guide is the skill and method of a leader that induces voluntary participation and cooperation from participants to achieve the objectives or goals of a program [19]. The styles of such leadership include transformational leadership, transactional leadership, and servant leadership. Transformational leadership involves leaders presenting the hopes and goals expected of members and encouraging them to work together to achieve those goals, thereby bringing about performance through changes in attitudes and the establishment of values [20]. Transactional leadership is a form where leaders use rewards or regulations for certain outcomes to motivate subordinates to meet their demands [21]. Additionally, servant leadership is a style where the leader does not become the center of power within the organization, regardless of the subordinates’ awareness, focusing on supporting subordinates [22]. The psychological well-being of participants in face-to-face sports activities, which boosts confidence, emotional stability, happiness, and satisfaction during physical activities, is increasingly recognized as vital for achieving life goals, interpersonal relationships, and self-actualization [23, 24]. However, research on the impact of varying leadership styles of Pilates instructors on participants’ psychological well-being remains scant, particularly when compared to disciplines such as Taekwondo and general physical education. This study seeks to fill this gap by examining the influence of Pilates instructors’ leadership styles on class satisfaction and participants’ psychological well-being. Additionally, this study aims to analyze and elucidate the relationship between Pilates instructors’ leadership styles and the psychological well-being and class satisfaction of participants.

## Methods

### Participants

The study focused on investigating 39 Pilates studios across South Korea. The target participants were 460 members attending Pilates classes, from which a final sample of 388 responses was obtained. We used purposive sampling, a non-probability method, to meet our research objectives. In our study, all participants had experienced face-to-face classes at Pilates studios. Additionally, the classes were conducted in small groups or with 10 to 20 participants per class. To accurately assess the instructor’s leadership, we did not limit the type of class, and it was specified that the classes conducted were typical of the instructor’s usual teaching.

In terms of the sociodemographic characteristics of the participants, the gender distribution was 300 females (77.9%) and 85 males (22.1%). The most represented age groups were those in their 20s and 30s, each accounting for 90 participants (23.4%), followed by individuals in their 40s with 74 participants (19.2%), 50s with 60 participants (15.6%), over 60s with 38 participants (9.9%), and teenagers with 33 participants (8.6%). The primary motivations for participating in Pilates were body management (diet) with 142 participants (36.9%), followed by posture correction (straightening) with 74 participants (19.2%), pain relief (rehabilitation) with 60 participants (15.6%), stress relief with 52 participants (13.5%), improvement of physical fitness with 44 participants (11.4%), and fostering social connections with 13 participants (3.4%). Regarding the duration of participation in Pilates, the majority had been involved for less than six months with 148 participants (38.4%), followed by six months to 1 year with 119 participants (30.9%), 1 to 2 years with 68 participants (17.7%), more than three years with 31 participants (8.1%), and 2 to 3 years with 19 participants (4.9%). In terms of frequency of participation, the most common was twice a week with 179 participants (46.5%), followed by once a week with 101 participants (26.2%), three times a week with 91 participants (23.6%), and four times a week with 14 participants (3.6%) (Table 1).

**Table 1:** Sociodemographic Characteristics. *N* = 388

Characteristics	Category	Frequency	Percentage (%)
Gender	Male	85 (21.14 ± 5.45 years)	22.1
	Female	300 (44.06 ± 13.01 years)	77.9
Age	Teens	33 (15.00 ± 2.05 years)	8.6
	20s	90 (24.86 ± 2.78 years)	23.4
	30s	90 (34.89 ± 2.97 years)	23.4
	40s	74 (44.79 ± 3.42 years)	19.2
	50s	60 (54.99 ± 2.70 years)	15.6
	60 and above	38 (66.40 ± 3.43 years)	9.9
Primary motivations for participating in Pilates	Posture Correction	74	19.2
	Body Shaping (Diet)	142	36.9
	Stress Relief	52	13.5
	Pain Relief (Rehab)	60	15.6
	Socializing	13	3.4
	Improvement of Ability	44	11.4
Exercise Participation History	Less than 6 months	148	38.4
	6 months − 1 year	119	30.9
	1 year − 2 years	68	17.7
	2 years − 3 years	19	4.9
	More than 3 years	31	8.1
Frequency of Participation	Once a week	101	26.2
	Twice a week	179	46.5
	Three times a week	91	23.6
	Four times a week	14	3.6

### Procedures and materials

#### Organization of the questionnaire

The measurement tool employed in this study was a questionnaire, structured into several distinct sections. Each section targeted a specific aspect relevant to the research objectives: sociodemographic characteristics, types of leadership, class satisfaction, and psychological well-being. This multi-faceted approach allowed us to holistically assess the impact of leadership styles on class dynamics and individual psychological states.

**Leadership Types**: The questionnaire on leadership types used in this study was modified and refined based on the scales from the research conducted by Lee, SH and Lee, YK. et al. [25]. The leadership types were organized into three subtypes: transformational leadership, transactional leadership, and servant leadership, to suit the context of this study.

**Class Satisfaction**: The class satisfaction questionnaire was adapted and refined for this study’s objectives based on the scale developed by Beard and Ragheb [26]. Specifically, it was structured around four sub-factors: educational, environmental, social, and physical.

**Psychological Well-Being**: The questionnaire for psychological well-being was adapted and refined for this study based on the “Psychological Well-Being Scale (PWBS)” developed by Ryff [27] and revised by Kim, MS et al. [28]. The questionnaire was structured around four sub-factors: positive relations with others, purpose in life, self-acceptance, personal growth, and autonomy.

All three aspects – leadership types, class satisfaction, and psychological well-being – were measured using a 5-point Likert scale, ranging from ‘strongly disagree’ (1 point) to ‘strongly agree’ (5 points), to gauge the responses accurately.

### Validity and reliability of questionnaires

#### Validity of the questionnaire

##### Content validity

This study examines the effects of Pilates instructors’ leadership types on class satisfaction and psychological well-being and aims to verify the direct and indirect effects between leadership types, class satisfaction, and psychological well-being. To ensure the robustness of the content validity, the questionnaire was initially drafted based on established frameworks and scales in leadership and psychological well-being [29, 30]. A preliminary validity test was conducted to ensure the appropriateness and suitability of the items. Subsequently, three expert meetings were held, involving a panel of five experts in sports psychology, leadership studies, and psychometric testing, who have over ten years of experience. These meetings facilitated significant revisions and adjustments to the questionnaire, guided by the experts’ feedback, which improved the relevance and cultural appropriateness of the items. The content validity was further confirmed by incorporating this feedback, thus tailoring the questionnaire to the needs of this study.

##### Construct validity

An Exploratory Factor Analysis (EFA) was conducted to preliminarily verify the construct validity of the survey tool used in this research, employing the Principal Component Analysis (PCA) method for factor extraction and Orthogonal Varimax Rotation for factor rotation. This EFA approach, including the choice to omit items with factor loadings below 0.5 and commonalities below 0.4, is supported by guidelines that advocate these thresholds to ensure significant factor loadings and shared variance among items [31, 32]. The Kaiser-Meyer-Olkin Measure of Adequacy (KMO) was applied, with values above 0.5 indicating suitability for factor analysis. The sample used for this EFA was distinct from that used in the final application of the questionnaire to avoid circular validation processes, as this approach is recommended to enhance validation rigor [33]. Before conducting our main study, we carried out a preliminary survey involving 50 participants over the course of approximately one month. This was done in order to assess the relationships among variables, participants’ understanding of the items, and the reliability of our instruments. Following this preliminary phase, we made necessary revisions and improvements to our survey questionnaire. The results of the EFA are presented in Tables 2, 3 and 4, demonstrating preliminary construct validity.

**Table 2:** Exploratory Factor Analysis of Pilates Instructors’ Leadership types

Number of Items	Servant	Transformational	Transactional
5	0.883	0.047	0.265
	0.868	0.121	0.221
	0.861	0.103	0.255
	0.846	0.173	0.317
	0.823	0.131	0.082
5	0.129	0.886	0.117
	0.13	0.835	0.058
	0.04	0.828	0.292
	0.182	0.821	0.208
	0.062	0.761	0.358
4	0.271	0.232	0.857
	0.243	0.208	0.855
	0.271	0.196	0.855
	0.293	0.319	0.773
Eigenvalues	4.03	3.734	3.361
% of Variance	28.788	26.668	24.01
Cumulative % of Variance	28.788	55.456	79.466
x2 = 5431.808, Kaiser-Meyer-Olkin MSA = 0.889, df = 105, sig = 0.000			

**Table 3:** Exploratory factor analysis of class satisfaction

Number of Items	Physical	Environmental	Educational	Social
6	0.878	0.178	-0.028	-0.101
	0.826	0.216	-0.017	-0.065
	0.786	0.171	0.278	0.021
	0.753	0.286	-0.286	-0.022
	0.74	0.29	0.221	0.147
	0.734	0.201	0.011	-0.143
4	0.394	0.861	-0.182	0.056
	0.385	0.789	0.169	-0.028
	0.086	0.776	0.151	-0.486
	0.350	0.752	-0.202	0.261
2	-0.093	0.026	0.822	0.174
	0.160	-0.054	0.720	0.011
2	-0.313	-0.359	0.072	0.746
	0.073	0.192	0.147	0.625
Eigenvalues	4.296	3.017	1.557	1.342
% of Variance	30.682	21.549	11.12	9.586
Cumulative % of Variance	30.682	52.231	63.351	72.937
x2 = 468.612, Kaiser-Meyer-Olkin MSA = 0.729, df = 91, sig = 0.000				

**Table 4:** Exploratory factor analysis results of Psychological Well-being

Number of Items	Personal Growth and Autonomy	Positive Interpersonal Relationships	Self-Acceptance	Purpose in Life
5	0.813	0.072	0.184	0.265
	0.752	0.208	0.082	0.347
	0.737	0.359	0.231	-0.221
	0.611	0.277	0.38	0.365
	0.581	0.249	-0.329	0.369
4	0.074	0.869	0.016	0.194
	0.224	0.754	0.063	0.344
	0.356	0.636	0.314	-0.125
	0.202	0.568	0.24	0.287
2	0.103	0.117	0.874	0.236
	0.367	0.162	0.784	0.119
2	0.186	0.131	0.152	0.775
	0.136	0.367	0.21	0.691
Eigenvalues	4.884	3.559	2.467	1.000
% of Variance	28.729	20.934	14.512	5.882
Cumulative % of Variance	28.729	49.663	64.175	70.058
x2 = 4150.050, Kaiser-Meyer-Olkin MSA = 0.867, df = 136, sig = 0.000				

#### Reliability of the questionnaire

A reliability analysis was conducted to verify the reliability of the measurement tools used in this study. Internal consistency reliability was assessed using Cronbach’s alpha coefficient when measuring the same concept with multiple items. Generally, a Cronbach’s alpha coefficient above 0.70 is considered reliable. The detailed results are shown in Table 5.

**Table 5:** Validate the reliability of a Questionnaire’s measurement variables

Separate	Factors	Number of questions	Cronbach’s α
Leadership types	Transformational Leadership	5	0.911
	Transactional Leadership	4	0.951
	Servant Leadership	5	0.937
	Total	14	0.943
Class Satisfaction	Social	2	0.766
	Educational	2	0.835
	Environmental	4	0.741
	Physical	6	0.713
	Total	14	0.901
Psychological well-being	Self-acceptance	2	0.864
	Personal growth and autonomy	5	0.799
	Positive interpersonal relationships	4	0.858
	Life in purpose	2	0.886
	Total	13	0.920

#### Measurement instrument precision for study participants

Descriptive analysis was conducted to assess the accuracy and consistency of the measurement tools used by our research subjects. This analysis helps us understand the distribution, central tendencies, and variability of the collected data, providing a foundational overview of the data characteristics. The mean and standard deviation are presented in Table 6. Descriptive analysis summarizes the basic features of the data collected in our study, giving simple summaries about the sample and the measures. It helps in understanding the distribution and central tendencies of the data collected from participants.

Precision, in the context of measurement tools, refers to the consistency and reliability of the instrument. While descriptive statistics alone cannot fully ascertain precision, they offer valuable insights when combined with other analyses. The mean values indicate the average response for each measurement tool, helping us understand where most participants’ responses are centered. Consistency in these values across different groups or samples can suggest stable measurement. The standard deviation provides information on the spread of the responses. Low standard deviation values indicate that participants’ responses are close to the mean, suggesting that the instrument consistently measures the same construct. Skewness and kurtosis measures help us understand the distribution of responses. Skewness indicates the asymmetry of the data distribution, while kurtosis indicates the peakedness. Values close to zero suggest a normal distribution, implying that the measurement tool is not biased and consistently measures across a range of responses.

Descriptive statistics provide an overview of the data and should be considered alongside other validation methods. These results support the validity of our survey instrument.

**Table 6:** Measurement tools for research subjects

Measurement Tool	Min	Max	Mean	Std. Deviation	Skewness	Kurtosis
Leadership Style	2.06	5	3.62	0.67	-0.04	-0.68
Transformational Leadership	2	5	3.81	0.76	-0.29	-0.58
Transactional Leadership	1.71	5	3.46	0.84	-0.08	-0.99
Servant Leadership	1.2	5	3.66	0.85	-0.52	0.42
Psychological Well-being	1.86	5	3.78	0.54	-0.24	0.18
Self-acceptance	2	5	3.84	0.59	0	-0.06
Personal Growth & Autonomy	1.8	5	3.8	0.66	-0.24	-0.44
Positive Relations	1.67	5	3.68	0.68	-0.27	-0.09
Purpose of Life	2	5	3.78	0.68	-0.09	-0.29
Class Satisfaction	2	5	3.74	0.55	-0.08	0.07
Educational Class Satisfaction	2	5	3.85	0.7	-0.42	-0.46
Social Class Satisfaction	1.5	5	3.49	0.67	0.49	-0.17
Environmental Class Satisfaction	1.5	5	3.84	0.66	-0.25	0.12

### Statistical analysis

The survey method for this study was the self-administration method, where respondents filled out the questionnaire themselves. We excluded from the analysis any questionnaires that were either incomplete or contained responses that did not meet our criteria. The statistical processing was conducted using the social survey statistical measurement program SPSS software (IBM SPSS Statistics, Version 25.0, Armonk, NY: IBM Corp) [34]. The specific analytical techniques for achieving the research objectives are as follows.

Firstly, sociodemographic variables such as gender, age group, purpose of exercise participation, exercise experience, and frequency of exercise participation were analyzed through frequency analysis and descriptive statistics. Secondly, to ensure the validity and reliability of the questionnaire items for the variables of leadership types, class satisfaction, and psychological well-being, exploratory factor analysis (EFA) and reliability analysis (Reliability Analysis) were conducted. Thirdly, the analysis of differences in general variables was conducted using Kruskal-Wallis’s test for between-group differences. Fourthly, the verification of multicollinearity among independent variables was based on the tolerance limit and the Variance Inflation Factor (VIF). It was determined that the tolerance values were more significant than 0.1, and all VIF values were less than 10. Multiple regression analysis was used to verify the relationships between class satisfaction, psychological well-being, and the relationship between class satisfaction and psychological well-being. In addition, a Pearson Correlation Analysis was conducted using Python to analyze the relationships between variables. The correlation matrix derived from this analysis was visualized as a heatmap using the seaborn library in Python (Python Software Foundation, Version 3.10) [35]. The Pearson Correlation Analysis was conducted to analyze the relationships between variables, focusing on the types of leadership exhibited by Pilates instructors, class satisfaction, and the psychological well-being of the participants. The magnitude of the correlations was interpreted using the following guidelines: 0.00-0.19 indicates a very weak correlation, 0.20–0.39 indicates a weak correlation, 0.40–0.59 indicates a moderate correlation, 0.60–0.79 indicates a strong correlation, and 0.80-1.00 indicates a very strong correlation.

## Results

**Fig. 1:**
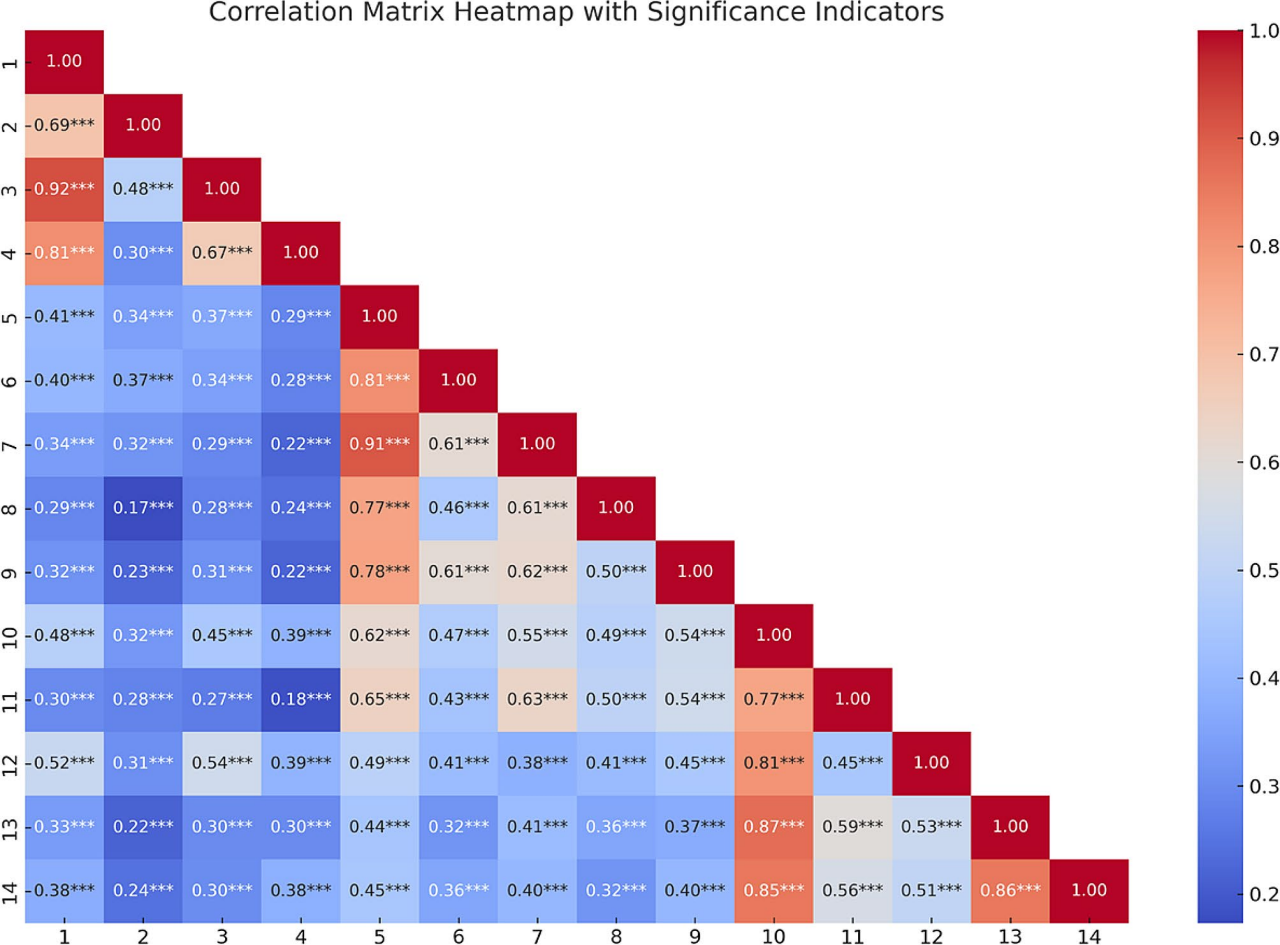
Correlation between measures in a study population. (1. Transformational Leadership, 2. Transactional Leadership, 3. Servant Leadership, 4. Self-acceptance, 5. Personal Growth and Autonomy, 6. Positive Interpersonal Relationships, 7. Purpose in Life, 8. Educational Satisfaction, 9. Social Satisfaction, 10. Environmental Satisfaction, 11. Physical Satisfaction, 12. Leadership, 13. Psychological Well-being, 14. Class Satisfaction)

The Pearson Correlation Analysis was conducted to analyze the relationships between variables, focusing on the types of leadership exhibited by Pilates instructors, class satisfaction, and the psychological well-being of the participants. As shown in the heatmap in Fig. 1, the correlation matrix illustrates the degree of correlation among the variables. The results of the Pearson Correlation Analysis reveal that the correlation between leadership types and psychological well-being was *r* = .407, indicating a moderate positive relationship. This suggests that more effective leadership styles are moderately associated with better psychological well-being among participants. The correlation between leadership types and class satisfaction was *r* = .483, also indicating a moderate positive relationship. This implies that effective leadership styles are moderately linked to higher class satisfaction among participants. Additionally, the correlation between psychological well-being and class satisfaction was *r* = .616, indicating a strong positive relationship. According to established guidelines [36, 37], correlations of 0.10 to 0.29 are considered small, 0.30 to 0.49 are considered moderate, and 0.50 and above are considered strong.

### The relationship between Pilates instructors’ leadership types and class satisfaction

The multiple regression analysis results for the impact of Pilates instructors’ leadership types on various dimensions of class satisfaction are summarized in Table 7. The analysis yielded significant models for each satisfaction dimension, indicating varying levels of explanatory power and significance of the different leadership styles.

For educational class satisfaction, transformational and transactional leadership positively influenced satisfaction significantly, whereas servant leadership did not show a significant effect. In the case of social class satisfaction, transactional leadership had a significant positive impact, while transformational and servant leadership did not have significant effects. When examining environmental class satisfaction, servant leadership was found to have a significant positive influence, unlike transformational and transactional leadership which did not show significant effects. Finally, for physical class satisfaction, both transformational and servant leadership significantly positively influenced satisfaction, with transactional leadership showing no significant effect.

**Table 7:** The impact of Pilates instructors’ Leadership types on Educational satisfaction, social satisfaction, environmental satisfaction, and physical satisfaction

Dependent Variable	Independent Variable	B	SE	Β	t	*p*	VIF
Educational Satisfaction	Constant	2.637	0.201	-	13.12	< 0.001	-
	Transformational Leadership	0.182	0.051	0.197	3.55	0.000	1.304
	Transactional Leadership	0.133	0.059	0.160	2.26	0.025	2.135
	Servant Leadership	0.015	0.054	0.018	0.28	0.782	1.806
F = 14.29, *p* < .001, R² = 0.1011, Adj R² = 0.0940							
Social Satisfaction	Constant	1.825	0.169	-	10.79	< 0.001	-
	Transformational Leadership	0.052	0.043	0.059	1.21	0.227	1.304
	Transactional Leadership	0.377	0.050	0.477	7.60	< 0.001	2.135
	Servant Leadership	0.043	0.045	0.055	0.95	0.345	1.806
F = 53.85, *p* < .001, R² = 0.2978, Adj R² = 0.2922							
Environmental Satisfaction	Constant	2.662	0.187	-	14.24	< 0.001	-
	Transformational Leadership	0.085	0.048	0.098	1.78	0.076	1.304
	Transactional Leadership	0.103	0.055	0.133	1.88	0.060	2.135
	Servant Leadership	0.137	0.050	0.177	2.73	0.007	1.806
F = 16.19, *p* < .001, R² = 0.1130, Adj R² = 0.1061							
Physical Satisfaction	Constant	2.469	0.184	-	13.42	< 0.001	-
	Transformational Leadership	0.122	0.047	0.139	2.50	0.010	1.304
	Transactional Leadership	0.008	0.054	0.010	0.15	0.880	2.135
	Servant Leadership	0.261	0.049	0.332	5.27	< 0.001	1.806
F = 24.88, *p* < .001, R² = 0.1638, Adj R² = 0.1572							

*B*: Coefficient, *SE*: Standard Error, *β*: Standardized Coefficient, *t*: t-statistic, *p*: *p*-value, *VIF*: Variance Inflation Factor

### The relationship between Pilates instructors’ leadership types and psychological well-being

A multiple regression analysis was conducted to investigate the impact of Pilates instructors’ leadership types on self-acceptance. The independent variables were the sub-factors of leadership types: transformational, transactional, and servant, while the dependent variables included self-acceptance, personal growth and autonomy, positive interpersonal relationships, and purpose in life. The results are summarized in Table 8.

The regression model for self-acceptance was significant, with transformational and transactional leadership having a significant positive impact, while servant leadership did not show a significant effect. For personal growth and autonomy, both transformational and transactional leadership had significant positive effects, whereas servant leadership did not show a significant effect. In the case of positive interpersonal relationships, transactional leadership had a significant positive impact, while transformational and servant leadership did not present significant effects. Lastly, for purpose in life, transactional leadership had a significant positive effect, whereas transformational and servant leadership did not show significant effects.

**Table 8:** The regression analysis results of the Relationship between Pilates instructors’ Leadership types and class satisfaction

Dependent Variable	Independent Variable	B	SE	β	t	*p*	VIF
Self-acceptance	Constant	2.441	0.161	-	15.16	< 0.001	-
	Transformational Leadership	0.207	0.041	0.267	5.03	< 0.001	1.304
	Transactional Leadership	0.105	0.047	0.151	2.22	0.027	2.135
	Servant Leadership	0.067	0.043	0.097	1.55	0.122	1.806
F = 27.39, *p* < .001, R² = 0.1774, Adj R² = 0.1709							
Personal Growth and Autonomy	Constant	2.491	0.186	-	13.39	< 0.001	-
	Transformational Leadership	0.201	0.048	0.231	4.23	< 0.001	1.304
	Transactional Leadership	0.110	0.055	0.141	2.01	0.045	2.135
	Servant Leadership	0.045	0.050	0.058	0.90	0.369	1.806
F = 18.46, *p* < .001, R² = 0.1269, Adj R² = 0.1200							
Positive Interpersonal Relationships	Constant	2.679	0.198	-	13.56	< 0.001	-
	Transformational Leadership	0.047	0.051	0.052	0.92	0.356	1.304
	Transactional Leadership	0.152	0.058	0.188	2.63	0.009	2.135
	Servant Leadership	0.080	0.053	0.099	1.51	0.132	1.806
F = 11.87, *p* < .001, R² = 0.0855, Adj R² = 0.0783							
Purpose in Life	Constant	2.678	0.195	-	13.72	< 0.001	-
	Transformational Leadership	0.091	0.050	0.101	1.82	0.069	1.304
	Transactional Leadership	0.197	0.057	0.244	3.45	< 0.01	2.135
	Servant Leadership	0.021	0.052	0.026	0.40	0.692	1.806
F = 14.78, *p* < .001, R² = 0.1043, Adj R² = 0.0972							

*B*: Coefficient, *SE*: Standard Error, *β*: Standardized Coefficient, *t*: t-statistic, *p*: *p*-value, *VIF*: Variance Inflation Factor

### The relationship between pilates participants’ class satisfaction and psychological well-being

A multiple regression analysis was conducted to determine the impact of class satisfaction types on self-acceptance among Pilates participants. The sub-factors of class satisfaction, including educational, social, environmental, and physical class satisfaction, were used as independent variables, while self-acceptance, personal growth and autonomy, positive interpersonal relationships, and purpose in life were used as dependent variables. The results are summarized in Table 9.

The regression model for self-acceptance was significant, with educational, social, and physical class satisfaction having significant positive impacts, whereas environmental class satisfaction had a negative impact. For personal growth and autonomy, educational and social class satisfaction were significant, while environmental and physical class satisfaction did not show significant effects. In terms of positive interpersonal relationships, educational and social class satisfaction had significant positive impacts, while environmental and physical class satisfaction were not significant. Lastly, for purpose in life, educational, social, and physical class satisfaction had significant positive impacts, whereas environmental class satisfaction had a negative impact.

**Table 9:** The regression analysis results of the relationship between pilates participants’ class satisfaction and Psychological Well-being

Dependent Variable	Independent Variable	B	SE	β	t	*p*	VIF	
Self-acceptance	Constant	1.944	0.178	-	10.94	< 0.001	-	
	Educational Satisfaction	0.258	0.047	0.309	5.49	< 0.001	1.626	
	Environmental Satisfaction	0.238	0.047	0.271	5.08	< 0.001	1.463	
	Educational Satisfaction	-0.172	0.080	-0.193	-2.14	0.033	4.152	
	Educational Satisfaction	0.187	0.076	0.212	2.45	0.015	3.841	
F = 33.57, *p* < .001, R² = 0.2611, Adj R² = 0.2533								
Personal Growth and Autonomy	Constant	1.261	0.177	-	7.11	< 0.001	-	
	Educational Satisfaction	0.535	0.047	0.571	11.39	< 0.001	1.626	
	Environmental Satisfaction	0.117	0.047	0.119	2.50	0.013	1.463	
	Educational Satisfaction	-0.020	0.080	-0.020	-0.25	0.802	4.152	
	Educational Satisfaction	0.038	0.076	0.038	0.50	0.618	3.841	
F = 67.14, *p* < .001, R² = 0.4141, Adj R² = 0.4079								
Positive Interpersonal Relationships	Constant	1.399	0.202	-	6.92	< 0.001	-	
	Educational Satisfaction	0.390	0.054	0.400	7.28	< 0.001	1.626	
	Environmental Satisfaction	0.246	0.053	0.241	4.62	< 0.001	1.463	
	Educational Satisfaction	0.061	0.091	0.059	0.67	0.502	4.152	
	Educational Satisfaction	-0.080	0.087	-0.078	-0.93	0.355	3.841	
F = 39.74, *p* < .001, R² = 0.2949, Adj R² = 0.2875								
Positive Interpersonal Relationships	Constant	1.231	0.192	-	6.40	< 0.001	-	
	Educational Satisfaction	0.418	0.051	0.430	8.19	< 0.001	1.626	
	Environmental Satisfaction	0.269	0.051	0.263	5.29	< 0.001	1.463	
	Educational Satisfaction	-0.182	0.087	-0.175	-2.09	0.038	4.152	
	Educational Satisfaction	0.180	0.083	0.176	2.18	0.030	3.841	
F = 52.92, *p* < .001, R² = 0.3577, Adj R² = 0.3510								

*B*: Coefficient, *SE*: Standard Error, *β*: Standardized Coefficient, *t*: t-statistic, *p*: *p*-value, *VIF*: Variance Inflation Factor

## Discussion

The primary aim of this study was to investigate the impact of Pilates instructors’ leadership styles on class satisfaction and psychological well-being among Pilates participants. Specifically, the study focused on how transformational, transactional, and servant leadership styles influence educational, social, environmental, and physical class satisfaction, as well as their subsequent effects on self-acceptance, personal growth and autonomy, positive interpersonal relationships, and purpose in life. The discussion is organized into several subsections to comprehensively address these aspects: the relationship between Pilates instructors’ leadership types and class satisfaction, the influence of leadership styles on psychological well-being, and the connection between class satisfaction and psychological well-being among Pilates participants.

### The relationship between Pilates instructors’ leadership types and class satisfaction

Higher transactional leadership in Pilates instructors increases class satisfaction, underscoring the value of rewarding participants upon achieving goals. This relationship is supported by the studies of Isebor [38] and Lim [39], while Park and Huh [40] and Lindberg [41] demonstrate that teachers’ behaviors directly affect students’ satisfaction, and perceived teacher-student congruence positively influences students’ satisfaction. Transformational leadership plays a significant role in educational class satisfaction, with the sub-factors of charisma and individual consideration positively impacting athletes’ satisfaction, as shown in the studies by Lim [39], Kim, Sim, and Lee [42], and Gorgulu [43]. The research also found that servant leadership significantly influences participants’ physical class satisfaction, as evidenced by the studies of Azadfada, Besmi, and Doroudian [44] and Muslih, Giyoto, Makruf, and Annisyaroh [45], highlighting the importance of a leadership approach that respects and supports participants. The study revealed no significant statistical relationship between environmental class satisfaction and leadership types, contrasting with Nezhad, Sani, and Andam [46], possibly due to the focus on well-equipped private studios, which may reduce the impact of environmental factors on leadership.

These findings suggest the need for training and education of Pilates instructors in setting positive and hopeful goals and providing services tailored to participants’ individual circumstances and characteristics. The findings underscore the importance of implementing training programs to cultivate professional attitudes and mindsets among instructors toward their clients.

### The relationship between Pilates instructors’ leadership types and psychological well-being

Self-acceptance, personal growth, and autonomy, sub-factors of psychological well-being among Pilates participants, were found to be significantly influenced by transformational leadership. In contrast, transactional and servant leadership did not show a significant impact. The findings suggest that when instructors display more transformational leadership, participants experience higher self-acceptance, personal growth, and autonomy. Transformational leadership is crucial in enhancing participants’ self-acceptance, personal growth, and autonomy. This leadership style focuses on recognizing and respecting individuals’ intrinsic values and potentials, helping participants embrace their identity and capabilities positively. A study examined the effects of transformational leadership on participants’ well-being and cognitive stimulation, revealing that transformational leadership decreased participants’ self-assessed cognitive stimulation and enhanced their well-being, contributing to a more positive perception of their situation and a higher valuation of their abilities and worth. Building on these findings, it is proposed that transformational leadership supports various dimensions of psychological well-being by virtue of its capacity for individual consideration and intellectual stimulation. Specifically, it is suggested that such leadership fosters self-acceptance and environmental mastery among followers by promoting a sense of autonomy and personal growth, ultimately leading to a sustainable enhancement of well-being that aligns with eudaimonia concepts [47].

Furthermore, a study in a learning program setting explored the role of peer instructors in constructing meaning, indirectly demonstrating how transformational leadership can foster individual autonomy and personal growth. The sub-factor of positive interpersonal relationships in Pilates participants’ well-being was not significantly impacted by transformational, transactional, or servant leadership styles. This could be attributed to a range of psychological and behavioral factors, as found in the health systems study [47], where individual stress responses and the quality of interpersonal relationships play a critical role. Additionally, as highlighted in the COPE HCW Trial [48], engagement levels and well-being are also influenced by various non-pandemic-related factors, including personal attitudes towards health interventions and the inherent dynamics within group settings, which can alter the effectiveness of leadership styles irrespective of external crises such as a pandemic.

The purpose in life was influenced by transactional leadership but not by transformational or servant leadership. Transactional leadership’s conditional rewards impact psychological well-being. Appropriate conditional rewards facilitate exercise participation and reduce disappointment from failure, providing positive motivation for participants through incremental goals and fair rewards. The influence of transformational, transactional, and servant leadership styles on positive interpersonal relationships among Pilates participants may be limited due to factors other than social distancing measures, such as the inherent individualism within exercise contexts. Previous studies, such as Huang [49], indicate that in fitness and recreational sports, leadership impact on interpersonal relationships can be overshadowed by personal goals and the solitary nature of the activity. This suggests that leadership styles may not be the primary driver in shaping relational dynamics in such settings.

Moreover, the distinct influence of transactional leadership on participants’ sense of purpose, as opposed to transformational or servant leadership, can be explained through the motivational framework provided by transactional leadership’s emphasis on clear rewards and recognition. This mirrors findings in educational contexts where transactional strategies significantly enhance students’ motivation by aligning rewards with performance objectives, thus supporting participants’ psychological well-being [50]. This reflects the theory that explicit incentives in transactional leadership can effectively motivate individuals towards specific achievements [51].

These insights suggest that while transactional leadership may be effective in contexts where direct rewards can be aligned with personal goals, the influence of leadership styles like transformational and servant leadership on interpersonal relationships may require a more integrative approach, especially in settings where individual pursuits predominate.

Appropriate conditional rewards can provide participants specific goals and rewards, encouraging exercise participation, reducing disappointment from failure, and offering positive motivation through achieving incremental goals. These findings indicate that the impact of leadership style on participants’ psychological well-being can vary depending on the situation. Particularly in abnormal situations like a pandemic, the influence of leadership style may differ from expectations, an essential consideration in adjusting leadership strategies.

### The relationship between pilates participants’ class satisfaction and psychological well-being

Educational satisfaction among Pilates participants was found to significantly influence personal growth and autonomy, sub-factors of psychological well-being, suggesting that higher educational satisfaction correlates with higher valuations of personal growth and autonomy. In contrast, social, environmental, and physical satisfaction did not significantly impact the sub-factors of psychological well-being. The findings reveal that higher educational satisfaction among Pilates participants fosters a greater emphasis on personal growth and autonomy. The observation that educational qualifications and satisfaction significantly contribute to multiple aspects of psychological well-being, such as personal growth and autonomy, is corroborated by a range of studies. Higher levels of education are associated with better mental well-being among young adult female university students, emphasizing the long-term benefits of educational attainment on psychological health [52]. Additionally, well-designed educational programs can enhance dimensions of psychological well-being, thereby promoting life satisfaction and self-regulation [53]. These findings underscore the importance of both educational qualifications and personal satisfaction in fostering an individual’s psychological development and autonomy. Furthermore, high satisfaction in educational activities can lead to growth in psychological well-being, affecting multiple dimensions such as autonomy, environmental mastery, and personal growth [54, 55].

Conversely, social, environmental, and physical satisfaction did not significantly influence the sub-factors of psychological well-being, highlighting the specific role of educational satisfaction in influencing certain aspects of psychological well-being and providing essential insights into understanding its overall impact.

Additionally, educational and social satisfaction among Pilates participants significantly impacted positive interpersonal relationships, a sub-factor of psychological well-being. Individuals more satisfied with their education and social experiences tend to value positive interpersonal relationships more. Environmental and physical satisfaction did not influence this sub-factor. This finding aligns with research indicating that positive relationships are the most influential on job satisfaction, indirectly supporting the impact of educational and social satisfaction on positive interpersonal relationships [56]. Other research has shown that perceived social support positively impacts psychological well-being and life satisfaction, bolstering the influence of social satisfaction on positive interpersonal relationships. Social support has been identified as a critical predictor of job satisfaction, with psychological well-being mediating this process [57, 58]. Educational and social satisfaction among Pilates participants also significantly impacted the sub-factor of life purpose. Higher educational and social satisfaction levels correlate with a greater sense of purpose and direction in life. The result that educational and social satisfaction significantly impacted life purpose suggests that participants are more likely to value their life’s purpose and direction. This alignment with previous research indicates that educational satisfaction positively affects individual life quality and job satisfaction, demonstrating a positive relationship between educational satisfaction and life satisfaction and positive interpersonal relationships [59]. Furthermore, research exploring the impact of social satisfaction on life purpose and direction in older adults suggests that social satisfaction can play a crucial role in enhancing an individual’s sense of purpose and direction. Educational achievements and social support positively influence life purpose and direction, supporting the positive impact of educational and social satisfaction on life purpose among Pilates participants [58, 60].

### Limitation

This study’s findings on the impact of Pilates instructors’ leadership styles on class satisfaction and psychological well-being are subject to several limitations that warrant further discussion. Firstly, the research was confined to a specific cultural context in South Korea, which may limit the generalizability of the results to other settings. Moreover, the reliance on self-reported measures could introduce response bias, a significant concern as these measures are susceptible to social desirability and recall biases. Recall bias, in particular, is well-documented in the literature. For instance, studies by Miller [61] and Van de Mortel [62] have shown how participants’ memories and subsequent reporting can be influenced by various factors, leading to potential inaccuracies in the data. Additionally, Embree and Whitehead [63] emphasized the challenges posed by response biases, including memory recall and social desirability, in self-reported drinking behavior.

One limitation of this study is the unbalanced sample in terms of sexes, with a significantly higher proportion of female participants. This imbalance could potentially affect the generalizability of the results, particularly to male participants. However, it is important to note that Pilates is predominantly practiced by women, as supported by previous studies. For instance, Segar et al. found that women are more likely to engage in Pilates and similar forms of exercise [64]. Furthermore, research by Brace-Govan indicates that women face specific barriers and motivations in physical activities, including Pilates, which may explain their higher participation rates [65]. These demographic trends can partially justify the observed sex discrepancy in our sample. Despite this justification, we acknowledge that the sex imbalance could still influence the outcomes and interpretations of our study. Future research should strive to include a more balanced sample to better understand the effects of Pilates across different sexes. Additionally, studies focusing specifically on male participants or using stratified sampling methods to ensure sex balance could provide more comprehensive insights into the influence of Pilates instructors’ leadership styles on class satisfaction and psychological well-being.

The cross-sectional design of the study further restricts the ability to establish causality. Confounding variables, such as participants’ prior experiences with Pilates, inherent personality traits, or concurrent participation in other forms of wellness activities, might have influenced the reported satisfaction and well-being, and were not controlled for in this study. This oversight could obscure the true effects of leadership styles on the outcomes measured. Additionally, focusing on urban participants may not capture the diverse experiences of individuals in rural or other differing environments, potentially overlooking how varying socio-economic and cultural backgrounds could affect class dynamics and satisfaction. Limiting the investigation to only three leadership styles may also fail to capture the full spectrum of possible influences, as other relevant styles or hybrid approaches could provide different insights into the leadership-effectiveness relationship.

Furthermore, the absence of Confirmatory Factor Analysis (CFA) in this study is a significant limitation. CFA could have provided a more rigorous test of the measurement model, ensuring that the constructs measured align with the theoretical expectations. Future research should incorporate CFA to validate the factor structure of the instruments used, enhancing the reliability and validity of the findings.

The purpose of our study was to investigate the health and exercise attitudes of Pilates participants. To this end, we surveyed over 300 Pilates participants. The sample size was based on a convenience sample, a method widely used in the initial stages of research. This choice of sample size, as observed in studies by Lausen et al. [23] and Bernardo [24], is a common approach in Pilates research. Although our sample does not perfectly represent all Pilates participants, it was sufficient to collect preliminary data and derive statistically significant results. This method was chosen considering the limitations of our resources and time constraints.

Future research should address these limitations by expanding the cultural contexts examined, utilizing objective measures to complement self-reported data, incorporating longitudinal designs to better establish causality, and exploring a broader array of leadership behaviors to fully understand their impact on class experience in diverse Pilates settings. Moreover, research should include both urban and rural participants as well as male participants to provide a more comprehensive understanding of how different environments influence class satisfaction and well-being.

In conclusion, while this study provides valuable insights into the impact of Pilates instructors’ leadership styles on class satisfaction and psychological well-being, it also highlights the need for further research to address the identified limitations and explore additional variables and contexts that may influence these outcomes.

## Conclusion

This study examines how Pilates instructors’ leadership types affect class satisfaction and participants’ psychological well-being. In order to achieve this goal, a survey was administered to 388 participants attending private Pilates studios in the metropolitan area. The conclusions of the research analysis are as follows:

Firstly, the leadership type of Pilates instructors influences class satisfaction. Specifically, transactional leadership, a sub-factor of leadership type, significantly affects social class satisfaction, transformational leadership influences educational class satisfaction, and servant leadership impacts physical class satisfaction. Secondly, the leadership type of Pilates instructors affects psychological well-being. Transactional leadership influences the sub-factor of life purpose in psychological well-being. In contrast, transformational leadership significantly impacts self-acceptance, personal growth and autonomy, and positive interpersonal relationships, sub-factors of psychological well-being. Thirdly, the class satisfaction of Pilates participants influences their psychological well-being. The sub-factor of social class satisfaction in Pilates participants class satisfaction affects the sub-factors of self-acceptance, positive interpersonal relationships, and life purpose in psychological well-being. In psychological well-being, educational class satisfaction impacts the sub-factors of self-acceptance, personal growth, autonomy, positive interpersonal relationships, and life purpose.

### Electronic supplementary material

Below is the link to the electronic supplementary material.

Supplementary Material 1

## Data Availability

The datasets used and analyses during the current study available from the corresponding author on reasonable request.
