# Supplementary material for: Exploring the influence of leadership styles on psychological well-being and satisfaction of Pilates classes clients
Source: BMC Sports Sci Med Rehabil. 2024 Jul 23;16:160. doi: 10.1186/s13102-024-00949-8 (PMC11265438; doi:10.1186/s13102-024-00949-8)
Supplement: Supplementary file 1 — Supplementary Material 1 [file 13102_2024_949_MOESM1_ESM.pdf]

Questionnaire (Translated version)

	Leadership types
1	My leader presents a vision for my future that motivates me.
2	My leader suggests new methods for tackling difficult problems.
3	I feel a sense of pride in exercising with my leader.
4	My leader teaches me how to view things with a creative perspective.
5	My leader makes clear what the distinct and important goals are.
6	My leader provides rewards commensurate with the effort I put into exercising.
7	My leader clearly distinguishes my performance standards in exercise.
8	My leader provides rewards corresponding to our achievements.
9	My leader shows me ways to achieve what I want.
10	My leader has an accurate awareness of their own weaknesses and strengths.
11	My leader invests a lot of time and effort for my growth/development.
12	Rather than being authoritarian, my leader exerts leadership through personal influence and persuasion.
13	My leader believes that dedicating themselves to the members is a fundamental role.
14	My leader willingly accepts criticism from others.
15	My leader helps me when I am facing difficulties.
16	My leader strives to maintain a balance between short-term and long-term perspectives within the center.
17	My leader works to enhance the sense of community among trainees within the center.

	Class satisfaction
1	I am satisfied when comparing myself to others.
2	I like almost all aspects of my personality.
3	I have pride and confidence in myself.
4	Looking back on my life, I am satisfied with the outcomes so far.
5	Life has been a continuous process of growth.
6	It is important to have new experiences.
7	I tend to express my opinions clearly.
8	I do not judge myself by the standards of others.
9	I am confident that my opinions are correct.
10	I enjoy having intimate conversations with family and friends.
11	My friends and I trust each other.
12	I have more friends than most people.
13	I try to implement the plans I have made.
14	I live my life with life goals.

	Psychological well-being
1	The provision of instructional materials by the leader aids in understanding the techniques.
2	The leader's demonstration is helpful for acquiring skills.
3	The leader's variety of teaching methods helps maintain interest and enthusiasm in the class.
4	The leader's feedback contributes to the improvement of skills.
5	Pilates aids in the development of social skills.
6	Pilates provides opportunities to meet a variety of people.
7	Pilates is beneficial for interpersonal relationships.
8	Pilates allows one to feel a sense of achievement.
9	Participating in Pilates classes is enjoyable in itself.

	Psychological well-being
10	Pilates enables one to experience a sense of accomplishment.
11	There is no discomfort in using equipment and supplies during Pilates classes.
12	The auxiliary facilities do not cause inconvenience in conducting the class.
13	The Pilates class environment is pleasant.
14	The location for the Pilates classes is appropriate.
15	Pilates brings vitality to life.
16	Pilates helps in the stabilization of mind and body.
17	Pilates enhances physical fitness.
18	Pilates is beneficial for maintaining health.
